# Supplementary material for: Physical activity promoting teaching practices and children’s physical activity within physical education lessons underpinned by motor learning theory (SAMPLE-PE)
Source: PLoS One. 2022 Aug 1;17(8):e0272339. doi: 10.1371/journal.pone.0272339 (PMC9342796; doi:10.1371/journal.pone.0272339)
Supplement: S1 Table — (DOCX) [file pone.0272339.s001.docx]

**Supplementary material 1. Linear Pedagogy curriculum: Object control skills Lesson**

|  | **Key stage 1**  Pupils should develop fundamental movement skills, become increasingly competent and confident and access a broad range of opportunities to extend their agility, balance and coordination, individually and with others. They should be able to engage in competitive (both against self and against others) and co-operative physical activities, in a range of increasingly challenging situations.  ***Pupils should be taught to:***  - Master basic movements including running, jumping, throwing and catching, as well as developing balance, agility and co-ordination, and begin to apply these in a range of activities.  - Participate in team games, developing simple tactics. |
| --- | --- |
| ***B3*: *Lesson No*** | **Lesson 1** |
| *Lesson Outcome* | Demonstrate mastery over underarm throw. |
| ***Desired outcome*** | To be able to perform an underarm roll at for accuracy while stationary and while moving. |
| ***Progression based on Gentiles’ taxonomy*** | **Foster children motor skills learning by increasing the difficulty of the task over the lessons using Gentile’s taxonomy:**  **Body: from no body transport 🡪 to body transport**  **Object: from no object 🡪 to manipulation of object**  **Motion: from object still 🡪 to object moving**  **Intertrial Variability: from no intertrial variability 🡪 to intertrial variability** |
| ***B8:*** ***Whole Class Task Activity*** | **Warm up**  The orchestra  Children must imitate the teacher who is the orchestra leader.   - Open and close arms on horizontal plane and clap hands. - Hands close to the ground and then up over the head. - Claps hands on the legs. - Alternate one clap on legs and one with hands. - Alternate one clap on the chest and one with hands. - Clap hands behind the back and on the front - Claps on the floor.   Alternate claps.  The teacher divides children in groups, each group will perform a different clap. When the director of the orchestra (teacher) gives the signal children start clapping.  **Drill 1**  Simplification of underarm roll  Demonstration: The teacher swings an arm from back to front as a pendulum and uses a verbal cue to guide the speed. Children are asked to say “swing back and swing forward” while performing the movement.  Subsequently, the teacher demonstrates how to bend and get close to the floor while swinging the arm:  “Step forward and caress the grass”.  Last the last demonstration the teacher includes a step forward in the action:  “Swing back step forward and caress the grass ”.  **Drill 2a**  After performing drill 1 one correctly each child receives a ball.  Child will repeat the drill one:  “Swing back step forward and caress the grass ”.  With a ball in their hand.  **Drill 2b**  Children repeat drill 2a throwing the ball to a goal.  Each child is responsible for one ball and must collect it after throwing it. The target might be placed close to a wall, so the ball does not roll far. Alternatively, children could work in pairs: a child could stand with leg open, and another child could roll the ball between the legs of the companion (goal).  Throw  Target  Ball  Child   \|  \| \| --- \|   **Drill 2c**  Same as drill 2b but children use different balls/ different targets. Possible gamification, the pair of children that scores the highest number of goals will win (set a precise distance).  **Drill 3**  Children are asked to walk towards a target and perform an underarm roll without stopping. Subsequently, children are be asked to run and perform an underarm roll towards a target while running.  The drill becomes a relay: the team that scores more goals wins. The rules are the following: only a child per team can run.  run   \|  \| \| --- \|   **Drill 4**  The teacher devides children in groups. Two children roll a ball in front of the rest of a group of children. The other children will try to hit the rolling ball using small balls.   \|  \| \| --- \|   **Game**  Children are provided with balls within a safe zone and they have to hit in the targets on the other side of the hall. However, other children will try to stop them by tagging them. The children that get tagged must come back to the safe zone before attempting to score a goal again. It is not possible to throw from the safe zone. Only underarm throw is valid.   \| Goal is not valid from here  Safe zone \| \| --- \|   **Alternative game**  Stuck in the mud  If children get tagged, they must roll their ball to a target. If they miss it, they are stuck and they must wait for a mate to free them. To free children who are stuck other children must roll a ball between their legs. If the task is too complex, the teacher removes the targets.   \|  \| \| --- \|   **Cool down**  Walking around the space, quietly. Take a seat. The teacher asks questions about the lesson. |
