# Supplementary material for: Physical activity promoting teaching practices and children’s physical activity within physical education lessons underpinned by motor learning theory (SAMPLE-PE)
Source: PLoS One. 2022 Aug 1;17(8):e0272339. doi: 10.1371/journal.pone.0272339 (PMC9342796; doi:10.1371/journal.pone.0272339)
Supplement: S2 Table — (DOCX) [file pone.0272339.s002.docx]

**Supplementary material 2. Nonlinear pedagogy curriculum: Invasion games lesson**
