# Supplementary material for: Physical activity promoting teaching practices and children’s physical activity within physical education lessons underpinned by motor learning theory (SAMPLE-PE)
Source: PLoS One. 2022 Aug 1;17(8):e0272339. doi: 10.1371/journal.pone.0272339 (PMC9342796; doi:10.1371/journal.pone.0272339)
Supplement: S3 Table — (DOCX) [file pone.0272339.s003.docx]

**Supplementary material 3. Table reporting inter-rater reliability results and the definition of each teaching practice**

|  | Definition |
| --- | --- |
| ***Lesson Context*** |  |
| Management- | Lesson time when students are not involved in physical education content, including transition, management, and break times. |
| Knowledge- | Lesson time focused on student acquisition of knowledge related to physical education. |
| Motor Content+ | Lesson time when students are engaged in activities involving motor content. |
| Fitness+ | Activities where the main purpose is to warm-up or train cardiorespiratory fitness, strength and flexibility. |
| Skill Practice+ | Activities where the main goal is to practice and improve movement skills. |
| Game Play+ | Activities where movement skills are applied in game situations. |
| Free Play+ | Time where children engage in play freely without the need of instruction. |
| Discovery Practice+ | Activities devoted to the exploration of different movement solutions to meet the task, to answer a question or to solve the problem proposed by the teacher. |
| ***Activity Context*** |  |
| Individual Activity+ | Students participate in an activity alone. |
| Partner Activity+ | Students participate in an activity in pairs. |
| Small Sided Activity+ | Students participate in activities divided into several small groups of not more than 5 children. |
| Large Sided Activity- | Students participate in activities divided into groups of more than 5 children. |
| Whole Class Activity+ | Students participate in activities as a large group and interact with each other to accomplish a goal. |
| Waiting Activity- | Activity where the majority of the children have to wait for their turn to play or participate. |
| Elimination Activity- | A game that involves the elimination of students from the activity. |
| Girls Only Activity+ | Activities that only include girls. |
| Children Off Task- | Time when one or more students are not engaged in the task proposed by the teacher. |
| ***Teacher Behaviours*** |  |
| Supervises+ | The teacher monitors the activity without intervening. |
| Instructs Single Child- | Teacher interacts with one student either verbally or nonverbally providing either instructions, demonstration or feedback. |
| Instructs Group- | Teacher interacts with a group of students either verbally or nonverbally providing either instructions, demonstration or feedback. |
| Instructs Class- | Teacher interacts with the whole class either verbally or nonverbally providing either instructions, demonstration or feedback. |
| Promotes PA+ | The teacher verbally promotes engagement in physical activity. |
| PA as Punishment- | When the teacher uses physical activity as a punishment for a misbehaviour. |
| Withholding PA- | The teacher removes one or more students from an activity, |
| PA Engaged+ | The teacher engages in physical activity together with the children. |
| Off Task- | The teachers engages in duties that are not related with the lesson. |
| ***Activity Management*** |  |
| Signalling- | The teacher signals students to stop. |
| Retrieving equipment Multiple access points  - | Students move or collect equipment from/to multiple areas. |
| Retrieving equipment One access point - | Students move or collect equipment from/to one area. |
| Interruption Public- | Teacher addresses an interruption or misbehaviour publicly. |
| Interruption Private- | Teacher addresses an interruption or misbehaviour privately. |

+ : Indicates that the teacher practice is theorised to foster engagement in moderate to vigorous physical activity based on results from Crotti et al. (2021).

- : Indicates that the teacher practice is theorised to hinder engagement in moderate to vigorous physical activity based on results from Crotti et al. (2021).

PA: Physical activity.
