# Supplementary material for: Physical activity promoting teaching practices and children’s physical activity within physical education lessons underpinned by motor learning theory (SAMPLE-PE)
Source: PLoS One. 2022 Aug 1;17(8):e0272339. doi: 10.1371/journal.pone.0272339 (PMC9342796; doi:10.1371/journal.pone.0272339)
Supplement: S4 Table — (DOCX) [file pone.0272339.s004.docx]

**Supplementary material 4. Pedagogical fidelity checklist**

| **Motor-learning-related items** | | | | | **Lesson Quartiles** | | | |
| --- | --- | --- | --- | --- | --- | --- | --- | --- |
|  | **Pedagogy A** | | **Sliding Scale** | **Pedagogy B** | **Q1** | **Q2** | **Q3** | **Q4** |
| 1 | To support learning of fundamental movement skills PE teacher/coach will manipulate the child’s movements through breaking the skill into component parts | | **1 2 3 4 5** | To support the emergence of functional movement solutions the PE teacher/coach will manipulate the task or environment but not the child |  |  |  |  |
| 2 | Children learn skill first in closed decontextualized environments then apply new skills in a performance environment | | **1 2 3 4 5** | Movements are always learnt in context (music, storytelling, scenarios or games) |  |  |  |  |
| 3 | All children transition between activities and task at roughly the same time | | **1 2 3 4 5** | Transitions may be whole class, group of children or individual child and involve manipulations of tasks and activities but could on the surface be quite minor |  |  |  |  |
| 4 | PE teacher/coach controls what equipment is used and when it is introduced to the children | | **1 2 3 4 5** | PE teacher/coach allows the children to choose which equipment to use and when they want to use it to help with finding solution to the task |  |  |  |  |
| 5 | Demonstrations of fundamental movement skill by adult or a competent child is preferred option in closed environment | | **1 2 3 4 5** | Demonstration are done in context to encourage children to explore unique performance solutions |  |  |  |  |
| 6 | The use of verbal instruction is prescriptive and focused on correct technical movement pattern | | **1 2 3 4 5** | Verbal instruction is short and not prescriptive, focused on the environment or task |  |  |  |  |
| 7 | Feedback is skill focused and prescriptive to learn ideal template | | **1 2 3 4 5** | Feedback is used to support alternative functional movement solutions |  |  |  |  |
|  | |  | Global items |  |  |  |  |  |
|  | | **Pedagogy A** | **Sliding Scale** | **Pedagogy B** |  |  |  |  |
| 1 | | Teacher prescribes children to perform fundamental movement skill or set of fundamental movement skills.  Children learn an optimal movement template or technique of a particular skill or series of skills | **1 2 3 4 5** | Teacher creates an environment for children to perform functional movement solutions through interaction with the environment and task.  Children learn to explore and interact with their environment to find functional solutions |  |  |  |  |
| 2 | | Lesson progression is through clear and linear structure, warm up, drills, game/performance and cool down | **1 2 3 4 5** | Lesson evolves through storytelling, scenarios or games |  |  |  |  |
