# Supplementary material for: Physical activity promoting teaching practices and children’s physical activity within physical education lessons underpinned by motor learning theory (SAMPLE-PE)
Source: PLoS One. 2022 Aug 1;17(8):e0272339. doi: 10.1371/journal.pone.0272339 (PMC9342796; doi:10.1371/journal.pone.0272339)
Supplement: Attachment — Submitted filename: Response to reviewers.docx [file pone.0272339.s006.docx]

**Response to reviewers**

**Reviewer 1**

| Comment 1  Thank you for addressing all of my previous concerns and comments. I believe the manuscript is much improved and nearly ready for publication. I have a two remaining issues, I'd like to see resolved prior to endorsing publication. | Answer  We would like to thank Reviewer 1 for the constructive and positive feedback. Our responses to specific comments are outlined below. |
| --- | --- |
| Comment 2  - Regarding your response to comment 5, I think part of the confusion stems from the use of the word, ‘responsiveness’ which generally implies a change. By measuring PA levels once, you really can’t measure responsiveness since we don’t know if these PA levels are responding to the intervention or if they are similar to baseline levels | Answer  To clarify the meaning of the word “responsiveness” we modified the sentence in lines 158-162 by adding the definition of responsiveness in brackets together with a relevant citation:  “Specifically, this study was designed to evaluate the implementation of the interventions and explore PA promoting teaching practices during PE lessons and participants’ responsiveness (that concerns the measurement of how far participants respond to, or are engaged by, an intervention (53)) in terms of children’s MVPA engagement, rather than to evaluate changes in these constructs from baseline to post-intervention.”  As suggested in the sentence in lines 158-162, the assessment of responsiveness in our paper falls under the definition: how far participants **are engaged by** an intervention.  The article we cited was:  Carroll C, Patterson M, Wood S, Booth A, Rick J, Balain S. A conceptual framework for implementation fidelity. Implement Sci. 2007 |
| Comment 3  - My confusion regarding your statement about your results suggesting that linear pedagogy could help achieve increased MVPA in PE was specific to your sentence on line 699 -700. Perhaps just make it clearer that although it was not demonstrated in this study, increases in time spent in motor content and teacher PA engagement may over time lead to increases in MVPA. It remains confusing why the text appears to be concluding that either pedagogy may lead to increased MVPA when your results do not support this. | Answer  To address any confusion in the interpretation of our findings we deleted the sentences that were in lines 699-700 and in lines 715-717 in our previous submission and we added the following sentence at the end of the “Teaching practices in pedagogies underpinned by movement learning theories” section in lines 726-729:  “Nevertheless, taking all the above findings together, the results suggest that Linear and Nonlinear pedagogical interventions both improve time allocated to movement competence practice but would need to adopt more PA promoting teaching practices to increase children’s MVPA in PE (70,80,81).”  We also deleted the words “Skill Practice and” in line 694 as “Skill Practice” was mentioned already in the previous sentence. |
